# Micro RNA-411 Expression Improves Cardiac Phenotype Following Myocardial Infarction in Mice

**DOI:** 10.1016/j.jacbts.2022.05.008

**Published:** 2022-08-17

**Authors:** Ardiansah Bayu Nugroho, Nicholas Stafford, Min Zi, Sukhpal Prehar, Ryan Potter, Dowan Kwon, Yulia Suciati Kohar, Efta Triastuti, Thuy Anh Bui, Elizabeth J. Cartwright, Delvac Oceandy

**Affiliations:** Division of Cardiovascular Sciences, Faculty of Biology, Medicine and Health, The University of Manchester, Manchester Academic Health Science Centre, Manchester, United Kingdom

**Keywords:** cardiac remodeling, heart failure, Hippo pathway, microRNA-411, myocardial infarction, cTnI, cardiac troponin I, CVEC, cardiac vascular endothelial cells, EdU, 5-ethynyl-2'-deoxyuridine, LAD, left anterior descending coronary artery, MI, myocardial infarction, miRNA, microRNA, MTT, 3-(4,5-dimethylthiazol-2-yl)-2,5-diphenyltetrazolium bromide, NFAT, nuclear factor of activated T cells, NRCF, neonatal rat cardiac fibroblast, NRCM, neonatal rat cardiomyocytes, PCR, polymerase chain reaction, PEI, polyethylenimine, pHH3, phosphohistone H3, qPCR, quantitative PCR

## Abstract

Induction of endogenous regenerative capacity has emerged as one promising approach to repair damaged hearts following myocardial infarction (MI). Re-expression of factors that are exclusively expressed during embryonic development may reactivate the ability of adult cardiomyocytes to regenerate. Here, we identified miR-411 as a potent inducer of cardiomyocyte proliferation. Overexpression of miR-411 in the heart significantly increased cardiomyocyte proliferation and survival in a model MI. We found that miR-411 enhances the activity of YAP, the main downstream effector of the Hippo pathway, in cardiomyocytes. In conclusion, miR-411 induces cardiomyocyte regeneration and improves cardiac function post-MI likely by modulating the Hippo/YAP pathway.

## Highlights

• miR-411 overexpression increases cardiomyocyte proliferation and survival.
• Direct myocardial injection of miR-411 improves cardiac function and remodeling following myocardial infarction in mice.
• Expression of miR-411 in cardiomyocytes increases YAP activity, the main downstream effector of the Hippo pathway.

## Visual Abstract

**Figure:**
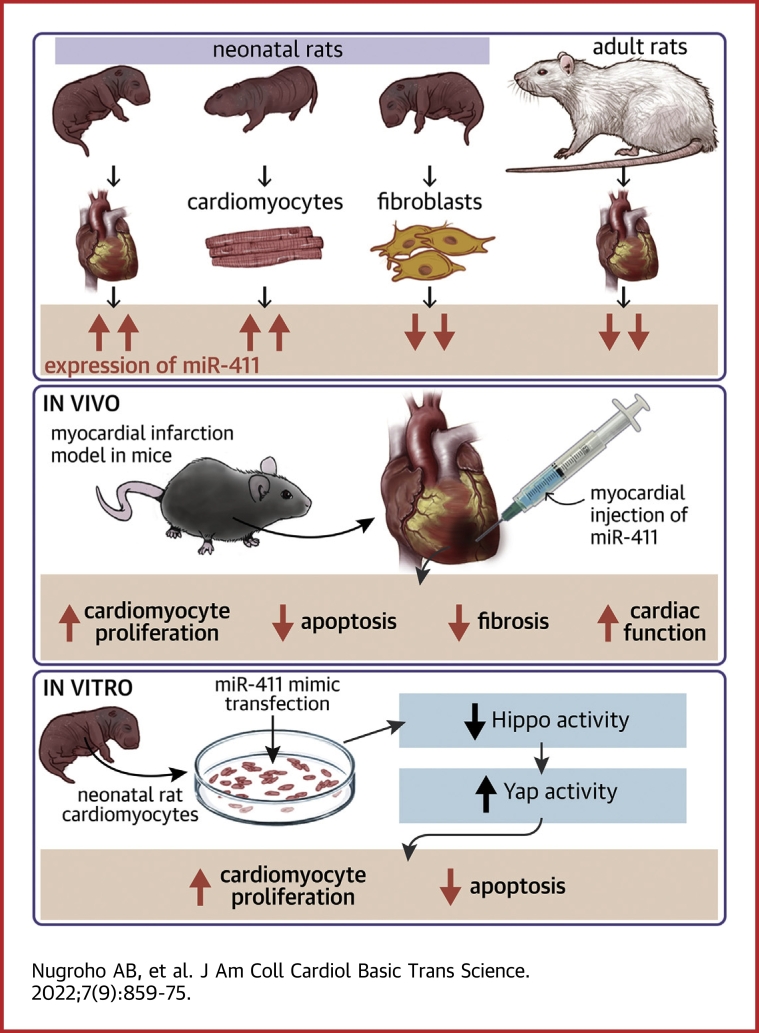

Heart attack or myocardial infarction (MI) is characterized by the death of large numbers of cardiomyocytes because of necrosis and apoptosis. As a response, the heart undergoes remodeling; which is characterized by the enlargement of remaining cardiomyocytes (hypertrophy), the replacement of dead cardiomyocytes with fibroblasts (fibrosis), and changes in cardiomyocyte metabolism and contractile function (reviewed by Shah and Mann^1^). Post-MI adverse remodeling is, at least in part, attributable to the limited capacity of adult cardiomyocytes to regenerate.^2^ However, recent advances in the understanding of cardiomyocyte cell cycle control have indicated that reactivating endogenous regenerative capacity in cardiomyocytes may become a promising approach to repair the infarcted heart.^3^^,^^4^

It is understood that cardiac growth during embryonic development mainly occurs through proliferation of cardiomyocytes (hyperplasia).^5^^,^^6^ In contrast, postnatal heart growth predominantly occurs via cardiomyocyte enlargement (hypertrophy) because terminally differentiated cardiomyocytes normally become mitotically inactive.^7^ Therefore, it is not surprising that the pattern of global gene expression in embryonic hearts is significantly different from that of postnatal hearts.^8^ This also raises the intriguing question of whether re-expression of developmental gene(s) can reactivate the ability of postnatal cardiomyocytes to proliferate.

MicroRNAs (miRNAs) are small noncoding RNAs that have strong effects on the regulation of gene expression. miRNAs have been shown as possible therapeutic targets for several pathologic conditions, including cardiovascular diseases.^9^ In the heart, several miRNAs have been demonstrated to modulate cardiac hypertrophy/heart failure,^10^^,^^11^ fibrosis,^12^ and cardiac pacemaker function.^13^^,^^14^ More interestingly, several studies have identified miRNAs that can induce cardiomyocyte proliferation.^15^, ^16^, ^17^ The possibility of inhibiting miRNA function using antagomirs or enhancing their biological activity using miRNA mimics makes miRNA an attractive therapeutic approach for cardiovascular diseases, including adverse heart remodeling and heart failure post-MI.

Based on this knowledge, we reasoned that miRNAs, which are highly expressed during cardiac development but not in the postnatal heart, might play an important role in mediating cardiomyocyte proliferation and, hence, can be targeted to induce cardiomyocyte regeneration. In this study, we were interested in investigating the role of hsa-miR-411-5p, hereafter referred to as miR-411, in mediating cardiomyocyte proliferation. A recent study indicated that miR-411 and its 5′ isomir are expressed in the heart and that their expressions are higher in embryonic tissues compared to newborn/adult tissues.^18^ miR-411 is also implicated in some cancer cells. Notably, in some studies, miR-411 has been shown to modulate cancer cell proliferation, although the effect seems to be conflicting among different cancer types. For example, in liver, bone, and lung cancer cells, miR-411 promotes cell proliferation,^19^, ^20^, ^21^, ^22^ whereas in ovarian, breast, bladder, and cervical cancers, miR-411 inhibits cell proliferation.^23^, ^24^, ^25^, ^26^ The fact that miR-411 is expressed in embryonic tissues and is involved in regulating the proliferation of several types of cancer cells prompts us to question whether this microRNA regulates cardiomyocyte proliferation.

## Methods

### In vitro experiments using neonatal rat cardiomyocytes

Primary neonatal rat cardiomyocytes (NRCMs) isolated from 2- to 3-day-old Sprague-Dawley rats were used to test the effects of miR-411 transfection. NRCMs were isolated and cultured according to protocols described in our previous publications.^27^^,^^28^ miR-411 and control miRNA (cel-miR-239b) were purchased from Horizon Discovery. Transfection was performed using Dharmafect reagent (Horizon Discovery), following the manufacturer’s instructions. Detailed protocols for NRCM isolation, culture, and miRNA transfection are available in the Supplemental Methods section.

### Quantitative polymerase chain reaction analysis

We used real-time reverse transcription polymerase chain reaction (PCR) analysis to examine the levels of native miRNA expression and the level of miR-411 following treatment with miRNA mimics. Total small RNA from cells or heart tissue was extracted using Trizol (Invitrogen), followed by purification with a Purelink miRNA isolation kit (Life Technologies) according to the manufacturer’s protocol. A total of 10 ng of miRNA were reverse transcribed with Taqman miRNA Reverse Transcription Kit (Thermo Fisher Scientific) using miRNA-specific stem loop primers (Taqman miRNA assays, Thermo Fisher Scientific). Then, 0.67 μL complementary DNA mixed with Taqman Universal Master Mix II and quantitative PCR (qPCR) primers (Taqman Small RNA Assay) were used for qPCR.

### Western blots

Western blot analyses were conducted to detect the expression levels of proteins or phosphoproteins from NRCMs or whole heart extracts. Western blots were performed following the protocols described in our previous publications.^27^^,^^28^ Detailed procedures and a list of antibodies used are available in the Supplemental Methods section.

### Analysis of cell proliferation and survival

We performed 5-ethynyl-2'-deoxyuridine (EdU) incorporation assay and analyzed the expression levels of the proliferation markers Ki67 and pHH3 to examine the effects of miR-411 transfection on NRCM proliferation, following protocols as described previously.^28^ In addition, we conducted 3-(4,5-dimethylthiazol-2-yl)-2,5-diphenyltetrazolium bromide (MTT) assay to assess cardiomyocyte survival following oxidative stress (H_2_O_2_ treatment). The detailed methods for proliferation analysis and MTT assay can be found in the Supplemental Methods section.

### Animal studies

Animal care and studies were conducted in accordance with the United Kingdom Animals (Scientific Procedures) Act and approved by the University of Manchester Ethics Committee. Mice were housed in a standard housing condition for laboratory animals and provided with food and water AD libitum. Researchers were blinded to treatments during data collection and analysis. Animal experiments are reported in compliance with the ARRIVE (Animal Research: Reporting of In Vivo Experiments) guidelines.^29^ Please see the Supplemental Methods section for details of the animal experiments.

### Signaling pathway screen analysis

We used luciferase reporters to initially screen signaling pathway(s) that might be regulated by miR-411. We analyzed the modulation of AP1, STAT3, YAP, Wnt, nuclear factor kappa B, and nuclear factor of activated T cells (NFAT) signaling using the luciferase reporter system. Detailed methods for the signaling pathway screen analysis and follow-up studies on Hippo pathway regulation can be found in the Supplemental Methods section.

### Statistical analysis

Data are presented as mean ± SEM. One-way analysis of variance, followed by Tukey’s post hoc test for multiple pairwise comparisons, was used to compare means in multiple group experiments. Student’s *t-*test was used to compare means in 2-group experiments. In the case that the data were not normally distributed, we used nonparametric tests (the Kruskal-Wallis test followed by Dunn’s multiple comparison or Mann-Whitney *U* test if it was a 2-group experiment). If nonparametric tests were used, the data are presented as the median with IQR. We used GraphPad Prism software, version 8.4.3 (GraphPad Software) for statistical analysis and data presentation. A *P* value of <0.05 was considered statistically significant.

### Data availability

All data supporting this study are provided in full in the Results section of this paper. All of the data are available from the corresponding author upon reasonable request. Expanded methods are available in the Supplemental Methods section.

## Results

### Expression of miR-411 in cardiomyocytes and in whole hearts

**Figure 1:**
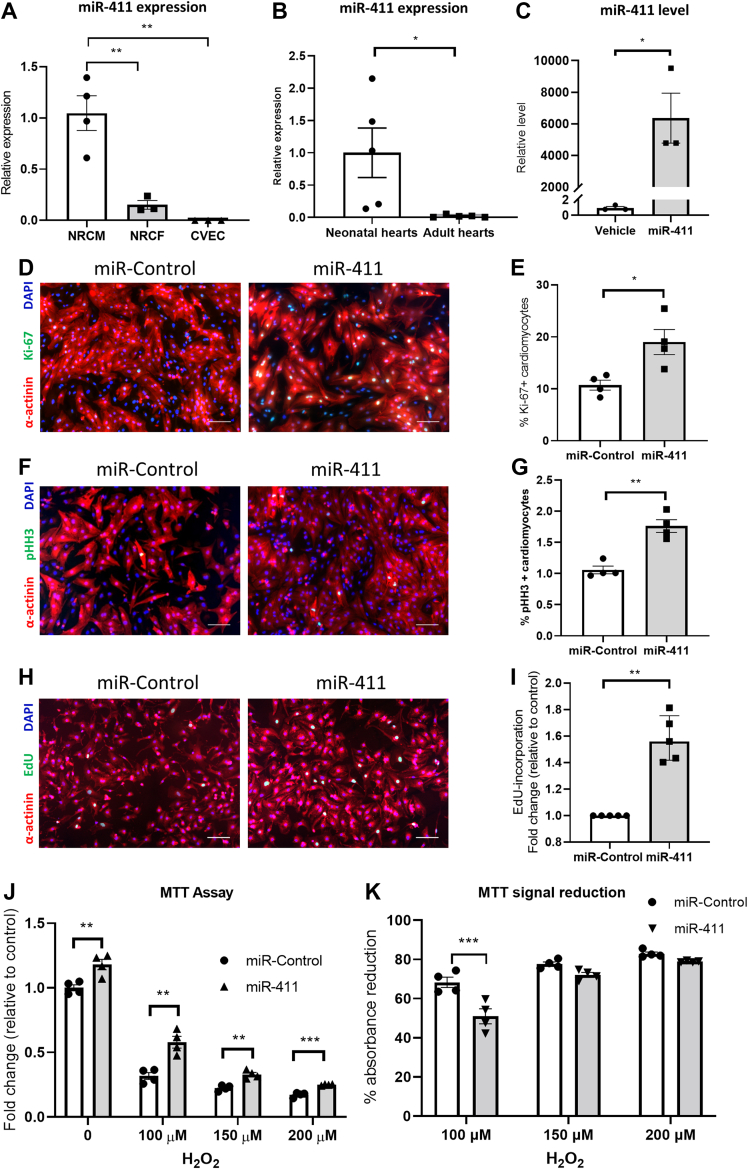
The Effects of miR-411 Overexpression on Cardiomyocytes **(A)** Endogenous expression of miR-411 was significantly higher in rat neonatal cardiomyocytes (NRCMs) than cardiofibroblasts (NRCFs) and cardiac vascular endothelial cells (CVECs) (n = 3 or 4 independent cell preps with a minimum of 3 replications in each prep). **(B)** The level of native miR-411 was markedly higher in neonatal rat hearts than in adult hearts (n = 5 in each group). **(C)** The level of miR-411 was significantly increased in NRCMs following transfection with miR-411 mimics (n = 3 independent experiments with a minimum of 3 replicates in each experiment). Expression of proliferation markers **(D, E)** Ki-67 and **(F, G)** pHH3 were significantly enhanced in NRCMs after miR-411 transfection (n = 4 independent experiments with a minimum of 3 technical replications each). **(H, I)** EdU incorporation assay showed increased cell proliferation in miR-411–treated NRCMs (n = 5 independent experiments). **(J)** Expression of miR-411 increased NRCM survival following treatment with H_2_O_2_ (100-200 μmol/L for 4 hours). **(K)** The reduction of MTT signal was significantly less in miR-411–expressing cells following treatment with 100 μmol/L H_2_O_2_, but not in cells treated with higher concentrations of H_2_O_2_ (n = 4 independent experiments with a minimum of 3 technical replicates in each experiment). Scale bars: 50 μm. Statistical tests used: **(A)** one-way analysis of variance followed by post hoc test for multiple pairwise comparisons, **(B, C, E, G)** Student’s *t-test*, **(I)** Mann-Whitney *U* test, and **(J, K)** multiple unpaired Student's *t*-test. ∗*P <* 0.05, ∗∗*P <* 0.01, ∗∗∗*P <* 0.001. DAPI = 4',6-diamidino-2-phenylindole; M = mol/L; MTT = 3-(4,5-dimethylthiazol-2-yl)-2,5-diphenyltetrazolium bromide.

We first investigated the expression of native miR-411 in cardiomyocytes and in the heart. We compared miR-411 expression in NRCMs, neonatal rat cardiofibroblasts (NRCFs) and rat cardiac vascular endothelial cells (CVECs). qPCR analysis showed that miR-411 was expressed in NRCMs, and its expression was significantly higher compared to that in NRCFs (Figure 1A). In contrast, we did not observe miR-411 expression in CVECs. We then analyzed the level of miR-411 expression in neonatal and adult rat hearts. miRNA-enriched RNA was isolated from 3-day-old rat neonate hearts and fully grown adult rat hearts (12 weeks old). We found that miR-411 expression was significantly higher in neonate hearts compared to adult rat hearts (Figure 1B).

### miR-411 transfection induces cardiomyocyte proliferation

To address the question of whether miR-411 is involved in mediating cardiomyocyte proliferation, we transfected NRCM with miR-411 mimics and analyzed the effect on cardiomyocyte proliferation. qPCR analysis showed a high level of miR-411 following transfection with the miRNA mimics (Figure 1C). We then assessed the levels of cell cycle markers Ki-67 and phosphohistone H3 (pHH3) and performed an EdU incorporation assay at 48 hours following transfection. Ki-67 and pHH3 are markers of mitosis, whereas EdU incorporation is a specific marker of DNA synthesis. Data presented in Figures 1D, 1E, 1F, and 1G shows that following miR-411 transfection, NRCMs displayed significant increases in the number of Ki-67– and pHH3-positive nuclei. Meanwhile, EdU incorporation assay identified an increase in EdU-positive cells following treatment with miR-411 mimics (Figures 1H and 1I). Together, our results indicate that miR-411 overexpression induces proliferation in cardiomyocytes.

### miR-411 expression improves cardiomyocyte survival

To determine the effect of miR-411 on cardiomyocyte survival, we conducted an MTT assay in NRCMs overexpressing miR-411 or miR-control following exposure with H_2_O_2_ to induce oxidative stress. We found that NRCMs transfected with miR-411 displayed a significant increase in viability compared to control, both basally and after exposure to H_2_O_2_ at various concentrations (Figure 1J). Further analysis of the relative reduction of MTT signal following H_2_O_2_ treatment normalized to the basal MTT signal indicated that NRCM overexpressing miR-411 showed lower levels of cell death in response to stimulation with 100 μmol/L H_2_O_2_, but not after stimulation with higher concentrations of H_2_O_2_ (Figure 1K). These data show that miR-411 can significantly improve cardiomyocyte viability under oxidative stress.

### Effects of treatment with miRNA-411 in adult hearts in vivo

**Figure 2:**
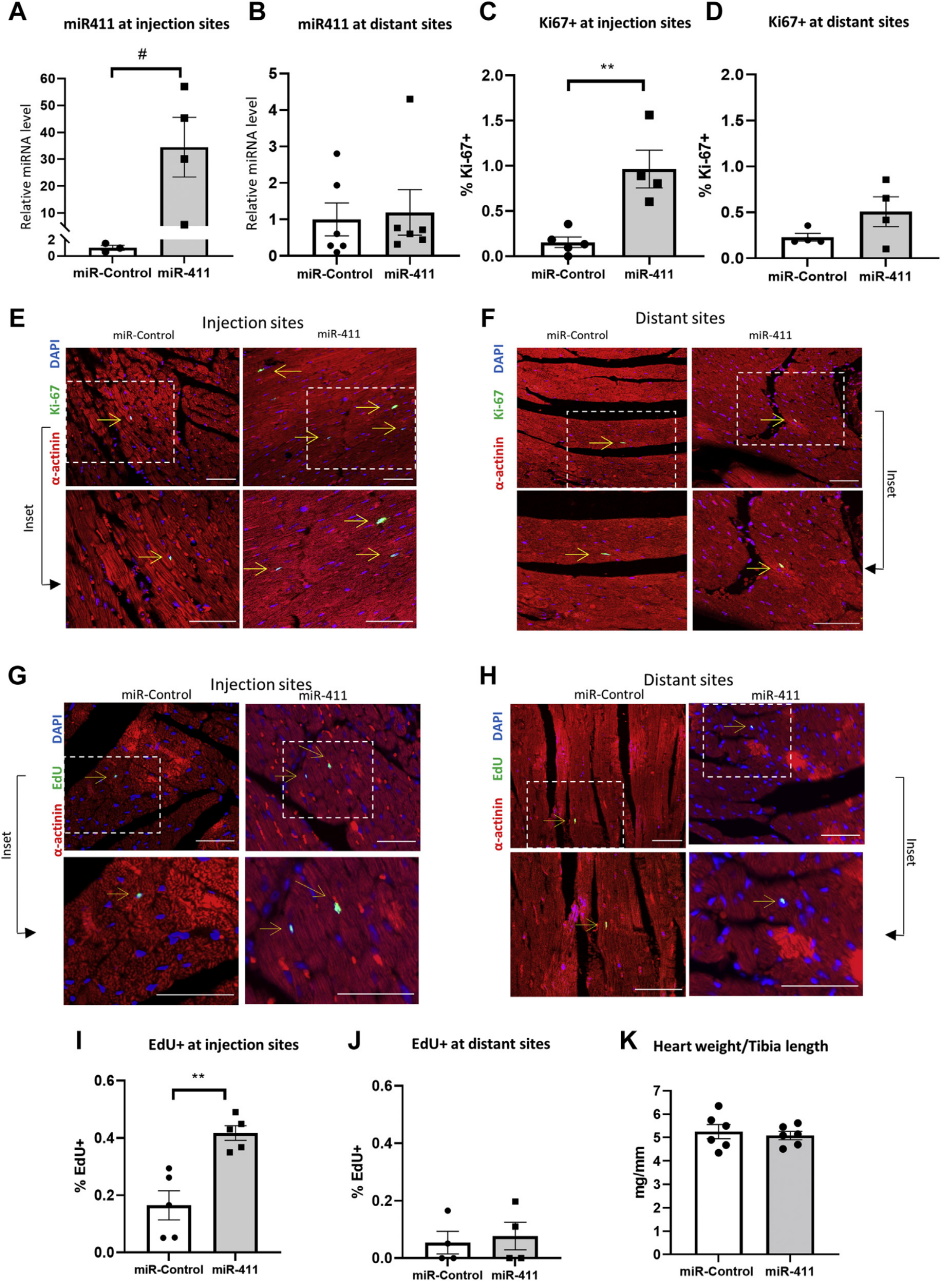
Direct Intramyocardial Injection of miR-411 Induces Cardiomyocyte Proliferation in Mice Wild-type C57Bl/6 mice were injected with miR-411/PEI complex or miR-control intramyocardially (5 μg/mouse). **(A)** Expression of miR-411 at injection sites and **(B)** miR-411 level at distant (noninjection) sites in the heart (n = 5 in each group). **(C to F)** Immunofluorescence analysis to detect Ki67-positive cardiomyocytes and quantification of Ki67^+^ cardiomyocytes at **(C, E)** injection sites and **(D, F)** distant sites. Arrows indicate Ki67^+^ cardiomyocytes. **(G to J**) Immunofluorescence detection of EdU incorporation and quantification of EdU^+^ cardiomyocytes at **(G ,I)** injection sites and **(H, J)** distant sites. Arrows indicate EdU^+^ cardiomyocytes (n = 4 or 5 in each group). **(K)** Analysis of the heart weight/tibia length ratio showed that there was no difference in heart size at 5 days following miRNA injection (n = 6 in each group). Statistical tests used: **(A to D, I to K)** Student’s *t-test*. ∗∗*P <* 0.01, ^#^*P* = 0.05. miRNA = microRNA; PEI = polyethylenimine.

We followed up the in vitro findings by analyzing the in vivo effects of miR-411 treatment in adult mouse hearts. The miR-411 mimics or control miR were mixed with nanopolymer polyethylenimine (PEI) as the delivery vector and then injected directly to the adult mouse myocardium (8 weeks old). We first analyzed the difference in the distribution of miR-411 mimics between heart segments at the injection sites and the apex of the heart (the distant sites) at 5 days postinjection. As expected, miR-411 level was markedly higher in the injection sites compared to hearts injected with control miRNA (cel-miR-239b) (Figure 2A). Interestingly, the level of miR-411 at the distant sites was similar to that of control hearts (Figure 2B). This suggests that up-regulation of miR-411 appeared to be spatially confined to the injection site at day 5 postinjection.

We went on to examine Ki-67 expression and EdU incorporation at day 5 post–miRNA injection. EdU was injected at day 3 post-miRNA treatment. We analyzed Ki-67 expression and EdU incorporation at both the injection and distant sites. Consistent with localized miR-411 distribution, we found that there was no difference in the number of Ki-67– and EdU-positive nuclei in the distant sites between miR-411–treated and control groups. However, we detected a significant increase in Ki-67– and EdU-positive nuclei in the injection sites in the miR-411 group when compared to control (Figures 2C to 2H). Our observations suggest that most of the EdU^+^ and Ki-67^+^ cells were also expressing the cardiomyocyte marker α-actinin, indicating that miR-411 treatment might induce adult cardiomyocytes to enter the cell proliferation cycle. However, we did not observe any change in cardiac size at 5 days following miR-411 injection, as indicated by comparable ratios of heart weight to tibia length, between mice injected with miR-411 and mice injected with control miRNA (Figure 2K).

### miR-411 treatment improves heart phenotype following infarction

**Figure 3:**
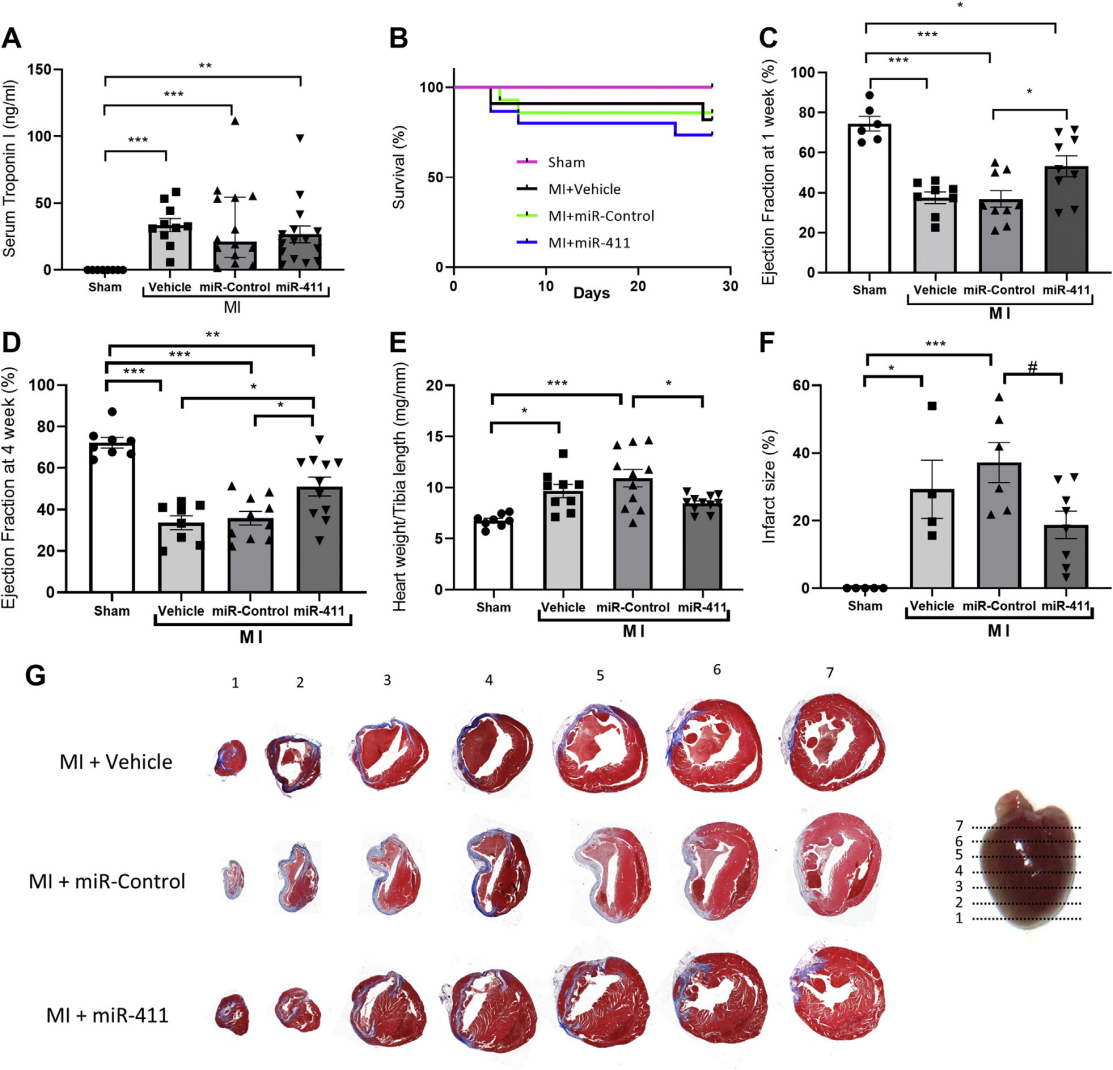
Treatment With miR-411 Improves Cardiac Function and Remodeling Following Myocardial Infarction Wild-type C57Bl/6 mice were subjected to MI and treated with direct intramyocardial injection of either miR-411, miR-control, or vehicle. **(A)** Levels of serum cardiac troponin I at 24 hours after MI. **(B)** Mice survival did not differ between MI groups at 4 weeks after MI. Cardiac function as indicated by **(C)** ejection fraction and **(D)** fractional shortening was significantly enhanced in the MI + miR-411 group compared to the MI + vehicle and MI + miR-control groups. **(E)** Cardiac hypertrophy, as indicated by the heart weight/tibia length ratio, was improved in the MI group after miR-411 treatment compared to the miR-control group. **(F, G)** Quantification of infarct size and representative Masson’s trichrome staining showing reduction of infarct size in the MI + miR-411 group (n = 8-11 in each group). Statistical tests used: **(A)** Kruskal-Wallis test followed by post hoc test for multiple comparisons. **(C to F)** One-way analysis of variance followed by post hoc test for multiple pairwise comparisons. ∗*P <* 0.05, ∗∗*P <* 0.01, ∗∗∗*P <* 0.001, ^#^*P* = 0.05. MI = myocardial infarction; miR = microRNA.

The evidence so far indicated that miR-411 is able to stimulate neonatal and adult cardiomyocyte proliferation and improve viability. Therefore, we hypothesized that miR-411 treatment would be beneficial following MI through induction of cardiomyocyte regeneration and would promote survival. To address this question, we generated an MI model in 12-week-old wild-type mice (C57Bl/6) by permanent ligation of the left anterior descending coronary artery (LAD). We then injected miR-411 mimic– or control miRNA–PEI complexes at regions adjacent to the LAD ligation site. We monitored cardiac troponin I (cTnI) levels in the plasma (Figure 3A) at 24 hours post–LAD ligation to validate the extent of MI. As expected, cTnI levels were significantly higher in groups subjected to LAD ligation compared to mice that underwent sham surgery. Importantly, there was no significant difference in cTnI level between miR-411–, miR-control–, and vehicle-treated groups, indicating a comparable level of MI between these groups (Figure 3A).

At the end of the experiment (4 weeks), the survival rates did not significantly differ between the MI animals treated with either vehicle, miR-control, or miR-411 (Figure 3B). However, echocardiography assessment showed that left ventricular ejection fraction was significantly improved in MI mice treated with miR-411 compared to those treated with vehicle or miR-control at both 1 week and 4 weeks post-MI (Figures 3C and 3D). Furthermore, analysis of cardiac structure and dimension demonstrated that miR-411 treatment significantly reduced the hypertrophic remodeling of the heart following MI, as indicated by a significant reduction in the ratio of heart weight to tibia length (Figure 3E).

**Figure 4:**
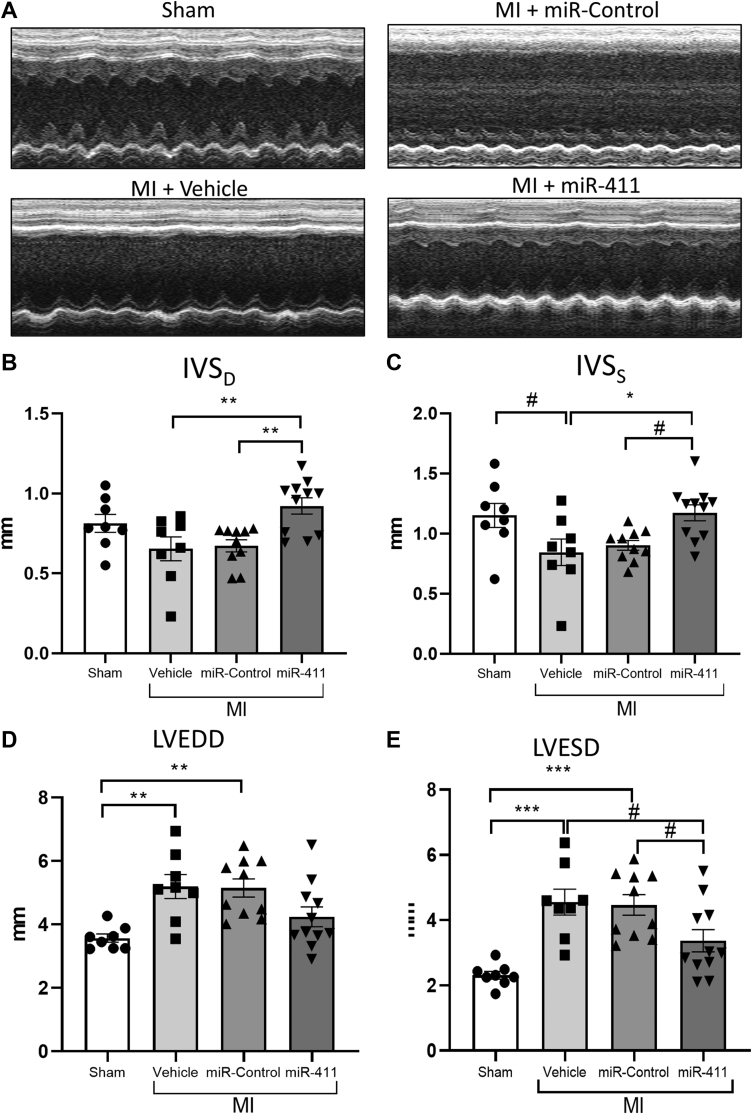
Echocardiography Analysis of Cardiac Morphology in Mice Following Myocardial Infarction **(A)** Representative images of M-mode echocardiography at 4 weeks following MI. **(B)** Analysis of interventricular septal wall thickness at diastole (IVS_D_) and **(C)** interventricular septal wall thickness at systole (IVS_S_) showing that treatment with miR-411 increased septal wall thickness following MI. **(D)** Analysis of left ventricular end diastolic diameter (LVEDD) and **(E)** left ventricular end systolic diameter (LVESD) suggested a trend of reduced left ventricular dilatation in MI mice treated with miR-411 (n = 8-11 in each group). Statistical tests used: **(B to E)** one-way analysis of variance followed by post hoc test for multiple pairwise comparisons. ∗*P <* 0.05, ∗∗*P <* 0.01, ∗∗∗*P <* 0.001, ^#^*P* = 0.05.

Analysis of Masson’s Trichrome–stained heart sections after MI demonstrated a significantly smaller infarct area in miR-411–treated mice compared to the control group (Figures 3F and 3G). In addition, miR-411–treated MI hearts displayed improved echocardiography parameters such as preserved left ventricular septum thickness and reduced dilatation compared to controls (Figures 4A to 4E).

**Figure 5:**
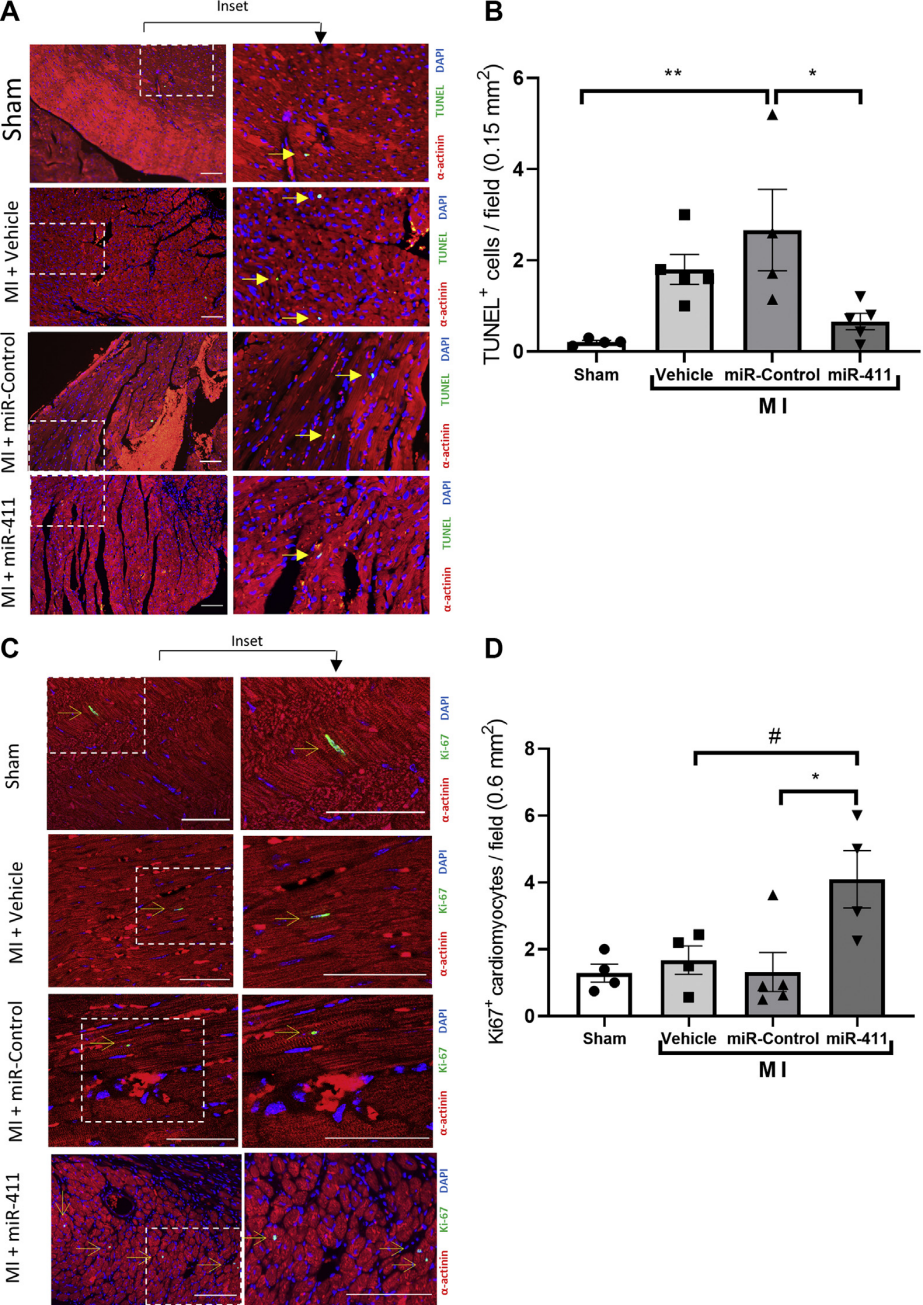
MiR-411 Overexpression Reduces Apoptosis and Increases Cardiomyocyte Proliferation in the Myocardial Infarction Model **(A)** Representative images of apoptosis analysis using TUNEL assay (arrows denote TUNEL-positive cells) and **(B)** quantification of TUNEL-positive cardiomyocytes in heart sections at 4 weeks after MI (n = 4 or 5 in each group). **(C)** Detection of Ki-67 expression was conducted by immunofluorescence analysis of cardiac tissue sections (arrows indicate Ki67-positive cardiomyocytes). **(D)** Quantification of Ki-67–positive cardiomyocytes suggests an increase in Ki-67–positive cells following miR-411 treatment (n = 4 or 5 in each group). Scale bars = 100 μm. Statistical tests used: **(B, D)** one-way analysis of variance followed by post hoc test for multiple pairwise comparisons. ∗*P <* 0.05, ∗∗*P <* 0.01, ^#^*P* = 0.05. MI = myocardial infarction; miR = microRNA; TUNEL = terminal deoxynucleotidyl transferase dUTP nick end labelling.

Furthermore, consistent with the protective effect of miR-411 treatment in vitro, the number of apoptotic cardiomyocytes, as indicated by Terminal deoxynucleotidyl transferase dUTP nick end labeling (TUNEL) assay, was significantly reduced in the miR-411 group compared to miR-control treated animals (Figures 5A and 5B). Apoptotic cells were mostly detected at the infarct border zone. Finally, to assess whether miR-411 induced cardiomyocyte proliferation post-MI, we analyzed Ki-67 expression in the heart sections. The number of Ki-67–positive cardiomyocytes was significantly increased at 4 weeks after MI in mice that had received miR-411 mimics (Figures 5C and 5D).

Taken together, our data demonstrate that injection with miR-411 improved cardiac structure and function following MI, likely by stimulating cardiomyocyte proliferation and improving cell survival.

### Identification of signaling pathway(s) regulated by miR-411

**Figure 6:**
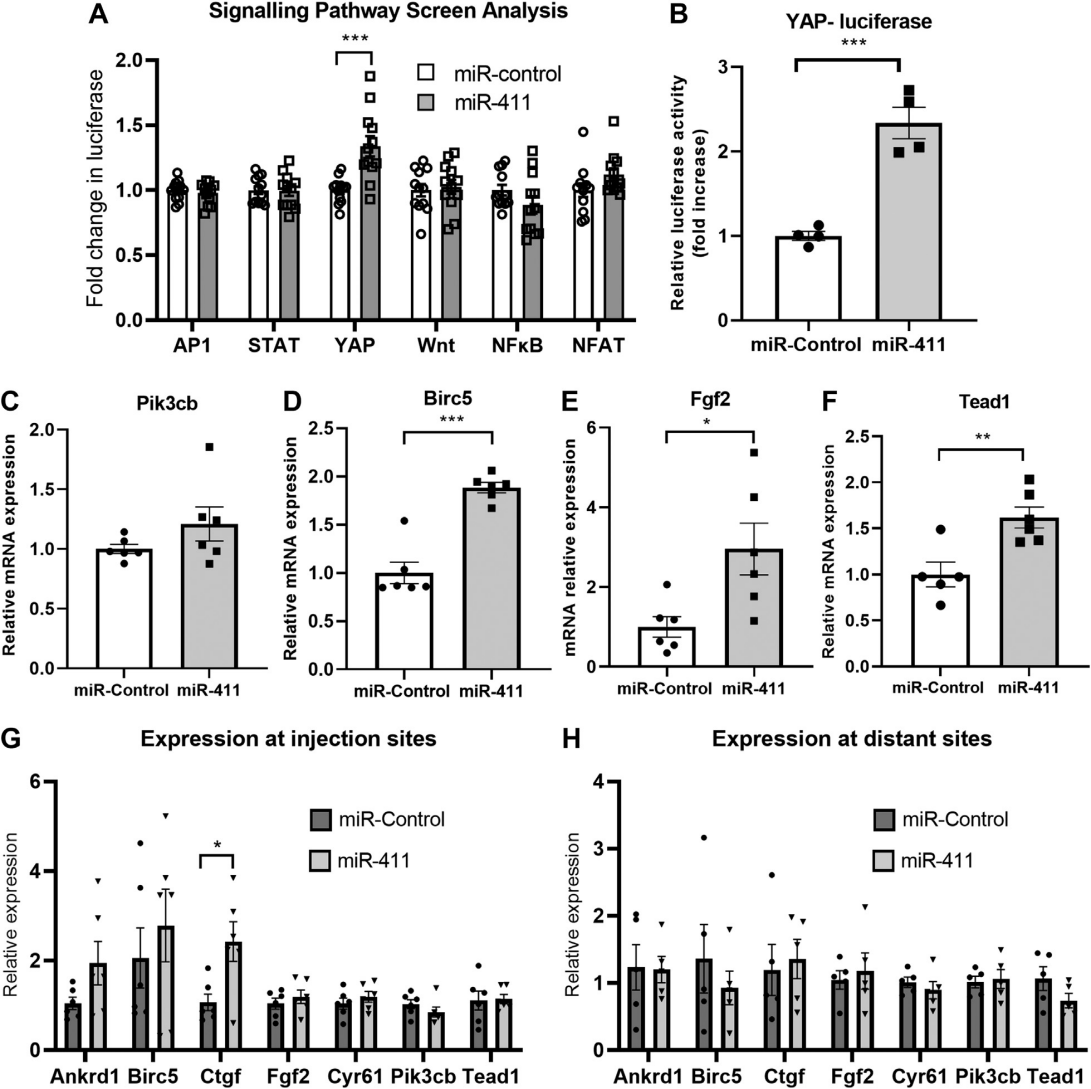
MiR-411 Modulates Hippo Pathway in Cardiomyocytes **(A)** Screen analysis using luciferase reporter constructs in H9c2 cardiac myoblast cell line revealed that miR-411 expression increased the activity of the YAP, but not Wnt, STAT3, nuclear factor kappa B (NFκB), nuclear factor of activated T cells (NFAT), or AP-1, signaling pathways (n = 12 in each group). **(B)** Confirmatory analysis using primary cardiomyocytes indicated that YAP activity was significantly enhanced in neonatal rat cardiomyocytes (NRCMs) after miR-411 transfection (n = 4 independent experiments with a minimum of 3 technical replications in each experiment). **(C to F**) Analysis of YAP-target gene expression, including **(C)** Pik3cb, **(D)** Birc5, **(E)** Fgf2, and **(F)** Tead1 in NRCM following transfection with miR-411 or control (n = 5 or 6 independent experiments in each group). **(G, H)** Analysis of YAP-target gene expression in mouse heart tissues following direct injection with miR-411. **(G)** Expression of Ctgf was significantly increased at injection sites; however, **(H)** no YAP-target genes examined exhibited elevated expression at distant sites. Statistical tests used: **(A)** multiple unpaired Student's *t*-test, **(B to F)** Student’s *t*-test, and **(G, H)** multiple unpaired Student's *t*-test. ∗*P <* 0.05, ∗∗*P <* 0.01, ∗∗∗*P <* 0.001.

To gain mechanistic insight into how miR-411 might regulate cardiomyocyte proliferation and survival, we screened the activity of a number of pathways commonly involved in these processes following miR-411 transfection in H9c2 cardiomyoblasts. H9c2 cells were transfected with luciferase constructs reporting the activity of either the β-catenin (Wnt pathway), nuclear factor kappa B, NFAT, YAP, STAT3, or AP1 signaling pathways. After 24 hours, cells were transfected with either miR-control or miR-411 mimics, and luciferase activity was measured 48 hours later. As shown in Figure 6A, miR-411 induced a significant increase in YAP luciferase activity, indicating a possible involvement in the regulation of YAP activity and/or the Hippo pathway. In contrast, miR-411 overexpression did not affect the activity of any other pathway examined.

### miR-411 modulates the Hippo pathway in cardiomyocytes

We followed up our data by testing the effects of miR-411 transfection in primary NRCMs Consistent with the screening analysis in H9c2 cells, we observed a significant elevation of YAP-luciferase activity in NRCMs after transfection with miR-411 mimics (Figure 6B), confirming that miR-411 might play an important role in modulating the Hippo pathway in cardiomyocytes.

The core elements of the Hippo pathway consist of kinases that mediate a phosphorylation cascade. Upon activation of the Hippo pathway, the main downstream effector YAP is phosphorylated, leading to its cytoplasmic retention and deactivation. In contrast, inhibition of the Hippo pathway leads to YAP dephosphorylation, nuclear translocation, and induction of its target genes’ expression.^30^ Therefore, we followed up our analysis on Hippo pathway modulation by assessing the level of known YAP target genes’ expression, such as Pik3cb, Birc5, Fgf2, and Tead1, in NRCMs following miR-411 transfection. We found a significant increase in the expression levels of Birc5, Fgf2, and Tead1 in miR-411–transfected cells, whereas there was no significant increase in the level of Pik3cb expression (Figures 6C to 6F).

To follow-up these findings, we examined the expression of YAP target genes in mouse heart tissues after intramyocardial injection with miR-411. As shown in Figure 6G, we detected a significant increase in Ctgf expression and a trend toward elevated Ankrd1 and Birc5 at the injection sites, whereas at distant sites, we did not observe any elevations of YAP-target gene expression (Figure 6H).

**Figure 7:**
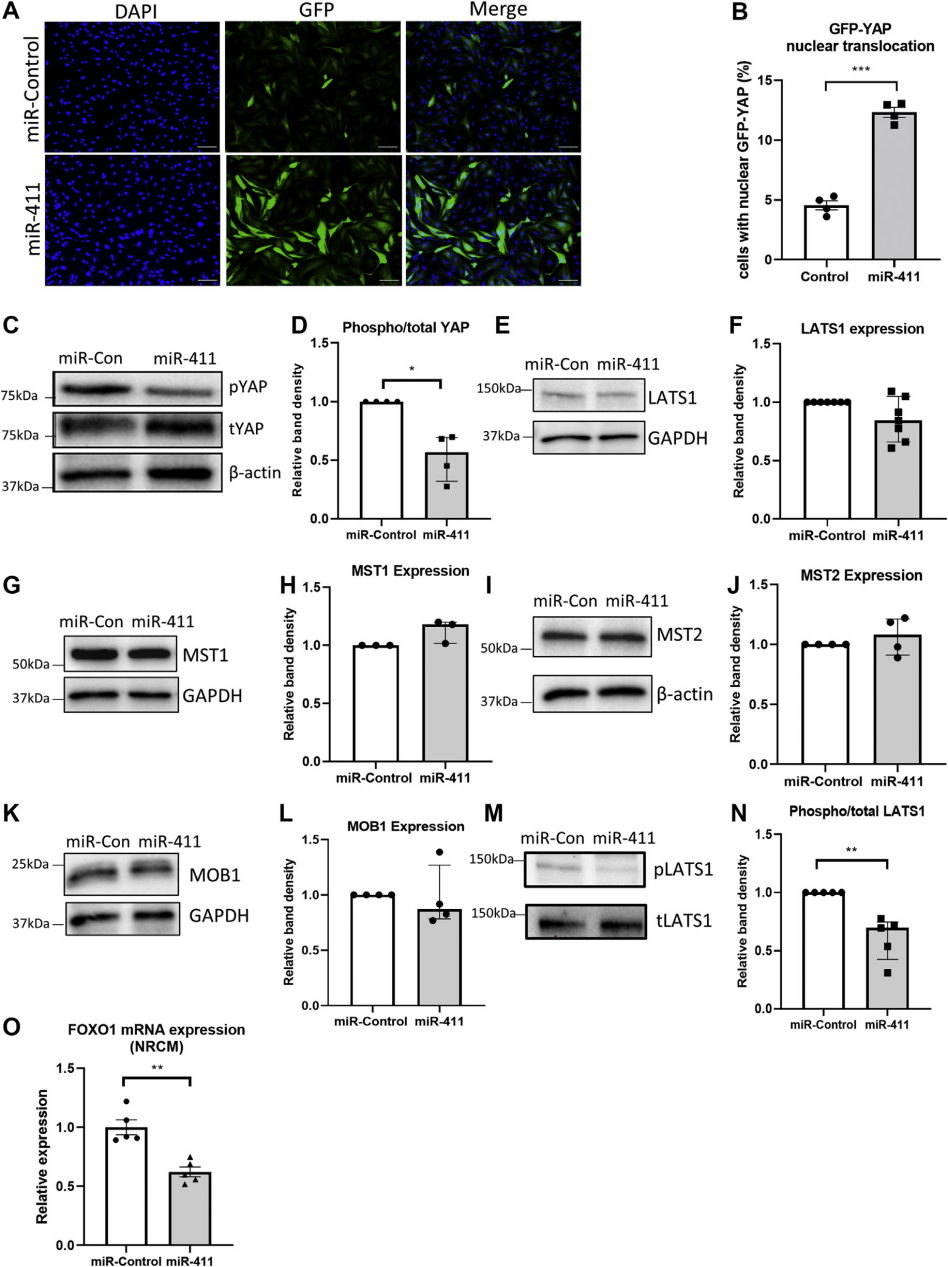
Regulation of the Core Components of the Hippo Pathway by miR-411 in NRCMs **(A)** Representative images of GFP-YAP nuclear translocation in NRCM-overexpressing miR-411 or miR-control. **(B)** Quantification of NRCMs with nuclear GFP-YAP showed that cells overexpressing miR-411 displayed a higher number of nuclear GFP-YAP (n = 4 independent experiments). **(C)** Representative Western blots and **(D)** quantification of band density showing the reduction of phosphorylated/total YAP in NRCMs after miR-411 transfection (n = 4 independent experiments). **(E to L)** Images of Western blots and quantification of band density displaying no changes in the expression levels of components of the Hippo pathway, including LATS1, MST1, MST2, and MOB1 in NRCMs after miR-411 treatment. **(M, N)** However, the level of phospho-LATS1 was significantly reduced in NRCMs transfected with miR-411 (n = 5 independent experiments). **(O)** Expression level of Foxo1 was reduced in NRCMs overexpressing miR-411, indicating that Foxo1 might be the target gene of miR-411 in cardiomyocytes (n = 5 independent experiments). Statistical tests used: **(B, O)** Student’s *t-test*. **(D, F, H, J, L, N)** Mann-Whitney *U* test. ∗*P <* 0.05, ∗∗*P <* 0.01, ∗∗∗*P <* 0.001. Con = control; miR = microRNA; NRCM = neonatal rat cardiomyoctes.

We went on by analyzing the YAP subcellular location using a GFP-YAP construct. As shown in Figures 7A and 7B, we observed a significantly higher proportion of nuclear YAP in cardiomyocytes expressing miR-411 compared to miR-control, indicating the nuclear translocation and, hence, activation of YAP following miR-411 overexpression.

Next, to investigate whether miR-411–dependent YAP activation in cardiomyocytes was caused by alteration in YAP phosphorylation, we assessed the level of phosphorylated/total YAP using Western blot. We found a significantly reduced phospho- to total YAP ratio in NRCMs transfected with miR-411 mimics (Figures 7C and 7D), suggesting that miR-411 may modulate YAP by altering the Hippo kinase cascade.

Because miRNAs are known to exert their biological effects primarily by reducing the expression of their target genes,^9^ we examined the expression level of the core components of the Hippo pathway. Surprisingly, we did not observe any changes in the expression of Hippo core kinases in NRCMs after transfection with miR-411 mimics (Figures 7E to 7L). However, we observed a significant reduction in LATS1 phosphorylation following miR-411 overexpression (Figures 6M and 6N), which was in agreement with the finding that YAP phosphorylation was reduced. Together, these data indicate that in cardiomyocytes, miR-411 modulates the Hippo kinase cascade without altering their total protein level, suggesting that the direct target of miR-411 might be outside of the Hippo core kinases, with subsequent effects on LATS1 and/or YAP activity.

### Foxo1 expression in cardiomyocytes was down-regulated following miR-411 expression

As shown in Figure 6, expression levels of the core members of the Hippo kinase cascade were not affected by miR-411 overexpression. Thus, we sought to find out if expression of known miR-411 target genes was affected following miR-411 expression in cardiomyocytes. One important and possibly relevant target gene was Foxo1. Foxo1 was previously identified as a direct target of miR-411 in lung cancer cells.^22^ Foxo1 is also known to interact with YAP in the cardiomyocyte nucleus,^31^ indicating its possible involvement in modulating the Hippo pathway. Therefore, we analyzed the level of Foxo1 expression in NRCMs following miR-411 transfection. Quantitative reverse transcription PCR analysis showed a significant reduction in Foxo1 expression in NRCMs transfected with miR-411 (Figure 7O), confirming that this gene might be a direct target of miR-411 in cardiomyocytes and might provide a link between miR-411 and Hippo pathway modulation.

## Discussion

The main finding of this study is that re-expression of a factor (ie, miR-411) that normally is expressed during embryonic organ development ameliorates cardiac remodeling and improves left ventricular function in a model of MI in mice. The mechanism likely involves the activation of YAP, which is the main downstream effector of the Hippo pathway.

### miR-411 expression improves cardiac phenotype post-MI

The significant improvement observed in miR-411–treated MI mice was likely caused by a reduction of infarct size, which consequently attenuated the cardiac remodeling process. This can likely be attributed to several factors, including increased cardiomyocyte proliferation and improved cell survival. The presence of proliferation marker Ki-67 and the increase in EdU incorporation in cardiomyocytes suggests an induction of cell cycle re-entry following miR-411 treatment.

Another important factor that may contribute to the reduced infarct size is the reduction of apoptosis. We observed that miR-411 treatment significantly reduced cardiomyocyte apoptosis at 4 weeks post-MI despite a comparable level of cardiac damage in the first 24 hours after MI, as suggested by the serum troponin level. In line with our findings, miR-411 has been reported to reduce cell apoptosis in several types of cancer.^21^ This suggests that miR-411 may protect cardiomyocytes from excessive apoptosis induced by hypoxia and oxidative stress during MI, which subsequently restricts the infarct size and limits the cardiac remodeling.

### miR-411 regulates the Hippo pathway in cultured cardiomyocytes

Our initial mechanistic analysis suggested that the Hippo signaling pathway might be implicated in the miR-411–dependent modulation of cardiomyocyte regeneration. The Hippo pathway consists of a complex of kinases and adaptor molecules that form a core kinase cascade. Activation of the Hippo kinase cascade leads to the inhibition of the main target effectors: Yes-associated protein (YAP) and transcriptional coactivator with PDZ-binding motif (TAZ).^30^ Indeed, recent studies have shown that inhibition of core components of the Hippo pathway^3^^,^^28^^,^^32^, ^33^, ^34^ and/or activation of the target effector YAP^35^, ^36^, ^37^ lead to the induction of cardiomyocyte proliferation and survival as well as improvement of cardiac remodeling in disease models, which subsequently leads to the repair of damaged hearts. Thus, our data showing that YAP activity was up-regulated following miR-411 overexpression are consistent with the widely accepted concept, ie, that induction of YAP activity is beneficial in stimulating cardiomyocyte proliferation and reducing adverse remodeling following MI.^35^, ^36^, ^37^

The antiapoptotic property of miR-411 might be, at least in part, caused by its ability to induce YAP activity. Increased YAP activity is associated with increased expression of antiapoptosis and prosurvival genes such as connective tissue growth factor (CTGF),^38^ cysteine-rich angiogenic inducer 61 (CYR61),^39^ and survivin.^40^ We also found that miR-411 reduced LATS1 phosphorylation. Because LATS1 is known as a strong proapoptotic kinase,^41^ this may contribute to the protective effect of miR-411 treatment.

Surprisingly, we did not observe any changes in the expression of the core components of the Hippo pathway following miR-411 expression in cardiomyocytes. However, our analysis showed that expression of Foxo1, which is a known target of miR-411,^22^ was significantly down-regulated in cardiomyocytes overexpressing miR-411. Foxo1 is a member of the forkhead box family of transcription factors.^42^ Most of the members of this superfamily of proteins are involved in the regulation of cancer cell development,^42^ but involvement in cardiac pathophysiology is also documented.^43^ It is known that the FoxO transcription factors are essential in the regulation of apoptosis, cell cycle, metabolism, and stress responses.^44^ Interestingly, Foxo1 is known to form an interaction complex with YAP in cardiomyocytes.^31^ This interaction resulted in YAP-dependent activation of Foxo1 transcriptional activity in mediating manganese superoxide dismutase (MnSOD) expression.^31^ However, it is not known whether Foxo1 itself modulates YAP transcriptional activity. Nevertheless, the facts that YAP and Foxo1 are closely linked and that miR-411 reduced Foxo1 expression prompt us to speculate that miR-411 induces YAP activity via modulation of Foxo1 expression. However, further studies still need to be done to confirm this mechanistic explanation.

### Direct intramyocardial injection for the delivery of miRNA

One key aspect of miRNA-based intervention is the strategy used to deliver miRNA to the target organ. This is crucial to achieve efficient therapeutic effects with minimal unwanted side effects. However, recent evidence shows that prolonged overexpression of proregenerative miRNAs using viral vectors could cause deleterious effects such as dilated cardiomyopathy and sudden cardiac death.^17^^,^^45^ To avoid this limitation, nonviral gene vectors such as polymer- and liposome-based delivery systems can be used as alternatives.^46^ Unlike using viral vectors, the effect of miRNA treatment using a nonviral system is transient and temporary. Previous investigations using lipid-based delivery systems reported that miRNA treatment can cause significant down-regulation of target genes, starting from 12 to 24 hours postinjection until 12 days postinjection.^16^^,^^47^^,^^48^ In this study, we demonstrated that nanopolymer PEI can successfully deliver miRNA mimics into the heart, as indicated by a significant increase in the miR-411 level 5 days post–intramyocardial injection. Interestingly, this increase was limited to the area near the injection site because miR-411 expression levels at distant sites in the myocardium were similar to controls. This suggests that intramyocardial injection of miR-411 mimics results in local overexpression only and may limit any off-target effects in other organs. Hence, this approach may become the preferred route of delivery in the future.

It is important to note that direct miR-411 injection may also affect noncardiomyocytes in the injection sites. In our model, we demonstrated significant effects of miR-411 injection in inducing cardiomyocyte proliferation and reducing cardiomyocyte apoptosis. This might be caused by the fact that in the heart, miR-411 is expressed predominantly in cardiomyocytes compared to other cell types such as fibroblasts or endothelial cells, and so miR-411 may have more significant biological functions in cardiomyocytes. However, it is still possible that miR-411 overexpression could affect the functions of other cell types, and further studies need to be conducted to address this issue.

### Study limitations

In this study we examined the phenotype of the mouse model at 4 weeks after MI. Although we did not see any adverse effect of miR-411 injection at this time point, there is a possibility of long term effects of miR-411 treatment that need to be evaluated. Nevertheless, this work has provided a valuable insight into the effect of miR-411 treatment in the acute phase of MI and has identified this micro RNA as a potential novel target for MI treatment.

## Conclusions

In conclusion, our study shows that overexpression of miR-411 in the heart can improve cardiac remodeling and function following MI. This will add to a growing body of evidence suggesting that miRNA treatments may provide a promising approach to control adverse cardiac remodeling post-MI.^15^^,^^17^^,^^48^ We also found that the regenerative effect of miR-411 might involve the modulation of the Hippo pathway. This is in agreement with recent data showing that many regenerative miRNAs are associated with the Hippo pathway.^17^^,^^49^

Perspectives **COMPETENCY IN MEDICAL KNOWLEDGE:** Following an acute MI, the heart undergoes adverse cardiac remodeling, encompassing processes including hypertrophy, dilatation, and fibrosis. These changes are at least in part caused by the inability of adult cardiomyocytes to regenerate following an infarction. It is hypothesized that re-expression of factors that are highly expressed during heart development may overcome this problem. We discovered that overexpression of miR-411 in cardiomyocytes induced cell proliferation and increased survival under stress. In an animal model of MI, we observed that treatment with miR-411 ameliorated adverse remodeling and improved heart function.

**TRANSLATIONAL OUTLOOK:** This study identified miR-411 as a candidate factor that can be targeted to control adverse heart remodeling following MI. As miRNA-based therapies have entered clinical trials for other diseases, it has been indicated that targeting of miR-411 is therapeutically feasible. Further studies using large animal models to confirm the therapeutic effects, to assess the long-term side effects/off-target effects, and to develop an effective delivery strategy will be needed to advance miR-411 toward translational application.

## Funding Support and Author Disclosures

This study was supported by British Heart Foundation Project and Programme grants [PG/17/78/33304 and RG/F/21/110055 to Dr Oceandy] and a Medical Research Council research grant [MR/P015816/1 to Dr Oceandy]. Dr Nugroho was supported by an Indonesian LPDP (Lembaga Pengelola Dana Pendidikan/Indonesia Endowment Funds for Education) PhD scholarship (S-476/LPDP.3/2016). All other authors have reported that they have no relationships relevant to the contents of this paper to disclose.
