# Supplementary material for: Micro RNA-411 Expression Improves Cardiac Phenotype Following Myocardial Infarction in Mice
Source: JACC Basic Transl Sci. 2022 Aug 17;7(9):859–75. doi: 10.1016/j.jacbts.2022.05.008 (PMC9617134; doi:10.1016/j.jacbts.2022.05.008)
Supplement: Supplemental Data [file mmc1.pdf]

SUPPLEMENTAL METHODS

In vitro analysis using neonatal rat cardiomyocytes (NRCM)

NRCM isolation and culture

Sprague Dawley rats were purchased from Charles River (UK). NRCM were isolated from 2-3 days old rat neonates according to a protocol described previously (1). In brief, the ventricles were separated from the atria, cut longitudinally into pieces, and dissociated enzymatically in artificial digestion solution buffer (116 mM NaCl, 1 mM NaH_2PO_4, 5.5 mM glucose, 5.5 mM KCl, 20 mM HEPES, 1 mM $MgSO_4$; pH 7.35) containing 0.6 mg ml^{-1} collagenase A (Roche) and 0.6 mg ml^{-1} pancreatin (Sigma). Digestion was performed in 7 min steps at 37°C in a shaking water bath, followed by passing the tissues through a pipette several times to detach the cells. The supernatant was collected after each step, passed through a 70-µm cell strainer, and supplemented with 2 ml FBS to neutralize collagenases. The pooled supernatant were centrifuged at 1,200 rpm for 5 min and the pellet resuspended in pre-plating medium (68% DMEM, 17% M199, 10% horse serum, 5% FBS and 2.5 µg ml^{-1}). Cells were plated on 10-mm culture dishes and incubated for 1 hour at 37°C for 1h to separate NRCM from cardiac fibroblasts. The supernatant, which mainly consisted of NRCM was collected and diluted at a concentration of $1x10^6$ cells ml^{-1} with plating medium (pre-plating medium with the addition of 1 µM BrdU (5-bromo-2-deoxyuridine)). Depending on the assay, NRCMs were seeded onto 6-, 24-well plates (BD Falcon Primaria) at an appropriate density and incubated at 37°C, 5% CO_2. The next day, the isolated cardiomyocytes were washed with PBS and kept in maintenance medium (80% DMEM and 20% Medium 199, 1% FBS, 2.5 µg ml^{-1} amphotericin B and 1 µM BrdU) at 37°C, 5% CO_2.

Rat primary cardiac endothelial cells

The rat primary cardiac microvascular endothelial cells were obtained from Generon. These cells were isolated from 6-8weeks of age Sprague-Dawley rat hearts. Cells were cultured in a

complete endothelial cell medium (Generon) supplemented with epidermal growth factor (EGF) and vascular endothelial growth factor (VEGF). Total micro RNA was isolated by using Trizol reagent (Invitrogen) followed by binding, washing, and elution steps with the PureLink miRNA Isolation Kit (Invitrogen).

miRNA transfection

Before transfection, all miRNA mimics (Horizon Discovery) were resuspended in 1x siRNA buffer (Thermo Fisher Scientific) at a concentration of 5 μM. Cardiomyocytes were seeded onto 6-well plates for protein extraction, 24-well plates for YAP luciferase or laminin-coated coverslips in 24-well plates for proliferation assay. After 24h incubation, cells were transfected with either control or miR-411 mimics at a final concentration of 25 nM according to the manufacturer's protocol. Briefly, both mimics and the transfection reagent (Dharmafect, Horizon Discovery) were each diluted with OPTI-MEM (Gibco) and incubated for 5 min at room temperature (RT). Both solutions were mixed, incubated for 20 min at RT, and loaded onto each well. Complete maintenance media was added into each well until the total volume was 500 μl for 24-well plates and 2 ml for 6-well plates. Cells were incubated for 48h at 37°C, 5% CO_2 for protein assay, Ki-67, and pHH3 immunofluorescence staining. For the EdU incorporation assay, 28h post transfection the culture medium was replaced with fresh medium containing 5 μM 5-ethynyl-2'-deoxyuridine (EdU, Life Technologies) and further kept for at least another 20h. miRNA transfection efficiency was assessed by qPCR detection of transfected miRNA

Western blots

Western blot analyses were conducted to detect expressions of proteins or phospho-proteins from NRCM or whole heart extracts. Western blots were performed following protocols described in our previous publications (1,2).

Protein lysates were extracted from NRCM mouse heart tissues using radioimmunoprecipitation (RIPA) buffer supplemented with proteinase inhibitors. Protein concentration was measured using a Pierce BCA protein assay kit (Thermo Fisher Scientific). Western blot was performed based on a standard protocol as described previously (1,3). 30 μg total protein from each sample were separated by 8-12% SDS-PAGE and transferred to PVDF membranes. Membranes were incubated overnight with primary antibodies: rabbit anti-LATS1 (Protein Tech, #17049-1-AP), rabbit anti-LATS2 (CST, #5888), anti-phospho-LATS1 (CST, #8654), rabbit anti-MST1 (CST, #3682), rabbit anti-MST2 (CST, #3952), anti-phospho-YAP (Ser127) (CST, #13008), anti-total YAP (Santa Cruz, #sc-101199), HRP-conjugated anti-α-tubulin (Abcam, #ab40742) and HRP-conjugated anti-β-actin (Abcam, #ab20272). The following day, membranes were washed three times, incubated with HRP-conjugated secondary antibodies anti-rabbit IgG (CST, #7074) or anti-mouse IgG (CST, #7076)) and visualized with ECL reagent (GE Healthcare) in a ChemiDoc XRS+ Imaging System (Bio-Rad).

Analysis of cell proliferation

Cell proliferation was investigated *in vitro* using immunofluorescence detection of cell cycle marker Ki-67 and mitosis marker pHH3 as well as by performing EdU incorporation assay. NRCMs were transfected with either miR-control (cel-miR-239b) or miR-411. At 48 hours post miRNA transfection, cells were washed with PBS several times, fixed with 4% paraformaldehyde for 15 min, permeabilized with 0.1% Triton X-100 in PBS for 10 min and blocked with 0.5% BSA for 1h at RT. Cells were then incubated with primary antibodies diluted in the blocking buffer, including rabbit anti Ki-67 antibody (Abcam, 1:100), Alexa Fluor 488-conjugated rabbit anti pHH3 antibody (Cell Signaling Technology, 1:100), and mouse anti α-actinin antibody (Sigma, 1:100) on a shaker overnight at 4ºC. The following day, cells were washed with PBS and incubated with secondary antibodies diluted in PBS

(FITC-conjugated anti rabbit and Alexa-Fluor 647-conjugated anti mouse antibody, Jackson ImmunoResearch, 1:100) for 1.5h at RT. Cell nuclei were identified by counterstaining with DAPI. Cells were washed with PBS several times, rinsed in distilled water, dried and mounted with mounting media (Vectashield, Vector Laboratories).

For the EdU incorporation assay we used a ClickIT EdU Imaging Kit Alexa Fluor-488 (Life Technologies) following the manufacturer's instructions. In brief, after incubation with blocking reagent, NRCMs were incubated overnight with diluted mouse anti α-actinin antibody (1:100), washed the next day and incubated with the respective secondary antibody for 2h. Cells were then washed again, incubated with the ClickIT reaction cocktails for 30 min and counterstained with Hoechst 33342 (Life Technologies).

From each biological replicate, Ki-67, EdU and pHH3 positive nuclei were counted from between 5-10 images (from 2 coverslips), or until the total number of NRCMs reached between 800 – 1000 cells. The percentage of Ki-67 or EdU or pHH3 positive cells was calculated by dividing the total number of Ki-67 or pHH3 positive cardiomyocytes with the total number of cardiomyocytes (α-actinin positive cells).

Analysis of cell survival in NRCM

Cardiomyocyte survival was assessed using the MTT (thiazolyl blue tetrazolium bromide, Sigma) assay. It was based on the reduction of yellow MTT salts to purple formazan crystals by oxidoreductase enzymes present only in viable cells. NRCMs seeded onto 24-well plates (BD Falcon Primaria Cell Culture Plates) and treated with either miR-control (cel-miR-239b) or miR-411 mimics for 48h. Cells were treated with hydrogen peroxide (H_2O_2) at concentrations of 100, 150, and 200 μM for 4h to induce oxidative stress. Purple formazan crystals were formed after 1h incubation with MTT working solution (5 mg/ml in PBS) and then dissolved with solubilization solution (0.1 N HCl in isopropanol). The absorbance was measured at 570 nm with a MultiSkan Ascent Plate Reader.

Animal experiments

In vivo direct intramyocardial injection of miRNA mimics

C57Bl/6 mice were purchased from Envigo (UK). Eight-week-old male C57Bl/6 mice were randomly allocated to receive an intramyocardial injection of miR-411 or miR-control (cel-miR-239b) at a dose of 5 µg per mouse heart. All miRNA mimics were purchased from Dharmacon (Horizon Discovery) and formulated with polyethylenimine (PEI) nanoparticles dissolved in 5% dextrose according to the manufacturer's instruction. The final injection volume per mouse heart was 25 µl with a 1:3 nucleic acid to PEI ratio. Mice were injected with buprenorphine 0.1 mg kg^{-1} subcutaneously as pre-operative analgesia. Induction of anesthesia was achieved by administering 5% isofluorane with O_2 at a rate of 1L min^{-1}. Mice were then intubated, mechanically ventilated (200 breaths per min, tidal volume 0.1 ml), and maintained at 3% isofluorane during surgery. The thorax was opened via an incision along the left parasternal border exposing underlying muscles; the pectoralis major muscle was lifted up and retracted whilst the now exposed pectoralis minor was dissected to expose the intercostal space. The 4th or 5th intercostal space was pierced and retracted to visualize the heart. The mimic solution was injected directly into the myocardium below the left atrial appendage using a 0.3 ml insulin syringe with a 30-gauge needle. The intercostal space, pectoralis muscles, and skin were then sutured with a 6-0 Prolene (Ethicon) and mice were extubated to establish normal breathing. They were placed in a prone position in an oxygenated chamber until they recovered fully from anesthesia. Following recovery, mice were housed in a 30ºC incubator before return to normal housing.

Myocardial infarction model

Myocardial infarction (MI) was induced in mice by permanently ligating the left anterior descending coronary artery (LAD) as previously described (3). Three months old wild type mice (C57Bl/6) were randomly allocated into four groups: sham, MI treated with vehicle, MI

treated with miR-control (cel-miR-239b), and MI treated with miR-411. After anesthesia and intubation, mice were laid in a supine position and fixed in place on a warming pad. A left sided thoracotomy was performed and the 4th – 5th intercostal space pierced and retracted to reveal the heart. The LAD artery was permanently ligated with 8-0 suture (Ethicon) below the left atrial appendage just above its bifurcation. The ligation was deemed successful once the left ventricular region distal to the ligation turned pale. Twenty-five microliters of previously prepared mix of miRNA mimics and PEI were injected into the anterior wall of the left ventricle. After ligation, the chest was closed and mice were housed in a 30°C incubator for a few hours before finally transferring to normal housing.

The level of serum cardiac troponin I (cTnI) was measured at 24 hours post-MI to confirm the presence and the extent of MI. At the end of experiments (4 week after MI) echocardiography analysis was performed to assess cardiac function and structure. Then the mice were euthanized and the hearts were extracted for histological, TUNEL and immunofluorescence analyses.

Cardiac Troponin I assay

Cardiac troponin I was measured to confirm the presence and the extent of MI. Blood samples were taken 24 hours post MI surgery via the lateral tail vein. In short, the mouse tail was anesthetized using topical cream (lidocaine 2.5%, prilocaine 2.5%). Mice were placed in restraint tubes on a warming pad to vasodilate the vein. After skin disinfection with betadine, a small transverse incision was made on the skin over the tail vein. Forty microliters of blood was collected and mixed with 3.2% sodium citrate to prevent coagulation. The samples were spun at 8000 rpm for 6 minutes to obtain the plasma.

Cardiac troponin I was measured using a high sensitivity mouse cTnI ELISA kit (Life Diagnostics) according to the manufacturer’s protocol. Plasma samples were diluted five times and standards were serially diluted to generate a standard curve. The samples and the

standards, together with 100 µl HRP conjugate, were dispensed into each well of a 96-well ELISA plate coated with anti-mouse cTnI antibodies and incubated for 1h at RT. The plate was washed and incubated with HRP substrate tetramethylbenzidine for 20 min at RT. Stop solution was added into each well turning initial yellow color into blue. The absorbance of each well was measured using a MultiSkan Ascent Plate Reader at 450 nm and plotted on the standard curve to calculate the cTnI concentration of each sample (ng ml^{-1})

Echocardiography analysis

To assess cardiac function at 4 weeks post LAD ligation, transthoracic echocardiography was performed using a VisualSonics Vevo 770 ultrasound fitted with a 30 MHz transducer, as previously described (1,3). Mice were anesthetized with 1.5% isofluorane and placed on a warming platform. M-mode images were acquired in both parasternal short and long axis views at multiple levels of the heart. Subsequently, measurements of the left ventricular internal diameter at end-systole and end-diastole, left ventricular anterior and posterior wall thickness, and interventricular septum thickness at diastole were obtained using the leading-edge method for a minimum period of 3 cardiac cycles. These values were used to calculate ejection fraction and fractional shortening using formulas described previously (2). Researchers involved in the analysis were blinded to the mouse treatment.

Histology, TUNEL and immunofluorescence analyses of heart tissue sections

At the end of MI experiments, mouse hearts were excised, briefly washed in PBS, and fixed in 4% paraformaldehyde overnight under agitation. The following day, the tissues were processed in a Leica ASP300 automated tissue processor overnight, embedded in paraffin and sectioned at 5 µm thickness using a rotary microtome (Leica 2255).

The scar size post infarct was assessed using Masson's trichrome staining. Tissue sections from 7 levels of the heart taken at a 500-µm interval were stained with Masson's trichrome using a standard protocol. Slides were imaged with a slide scanner (3D Histech Panoramic

250 Flash II) and analyzed with Pannoramic Viewer software. Infarct size was calculated by measuring midline fibrotic length detected in all levels of histological sections spanning from the apex to the base, divided by the sum of midline left ventricular circumferences from all levels of histological sections as described previously (4).

Cardiomyocyte proliferation *in vivo* was assessed using Ki-67 staining and EdU incorporation assay. Heart sections were deparaffinized in xylene for 30 min, and rehydrated with industrial methylated spirit at decreasing concentrations (100%, 90%, 75%) at room temperature. Antigen retrieval was performed by boiling the sections in sodium citrate buffer (10 mM Na citrate, 0.05% Tween-20, pH 6.0) for 25 min and letting them cool down to room temperature. Sections were rinsed three times with distilled water, permeabilized with 0.3% Triton-X in PBS for 30 min, and then blocked with 10% goat serum in PBS. For Ki-67 staining and EdU incorporation assay, slides were processed for immunofluorescence as described for cultured NRCM. Exclusively for the EdU assay, Tris-buffered saline (TBS) was used as washing buffer instead of PBS.

To assess apoptosis at 4 weeks post-MI, terminal deoxynucleotidyl transferase-mediated nick-end labelling (TUNEL) assay was performed on heart sections using a TUNEL staining kit (Roche) as described previously (2). Deparaffinized and rehydrated sections were incubated with 3% H_2O_2 for 15 min at room temperature, washed with PBS and treated with proteinase K for 15 min at 37ºC. For permeabilization, sections were incubated with 0.1% Triton-X and 0.1% sodium citrate in PBS for 15 min at 37ºC. Apoptotic nuclei were stained by incubating tissue sections with TUNEL working solutions for 1h at 37ºC in a humidified chamber. Sections were then blocked with 1% BSA in PBS for 1h at RT and stained with anti α-actinin antibody (Sigma, 1:100) overnight at 4ºC. The next day, sections were incubated with Alexa Fluor 647-conjugated anti mouse secondary antibody for 2h at RT, counterstained with DAPI, and mounted with Vectashield (Vector Laboratories). Positive cells were

quantified by measuring co-localization of DAPI signal with either Ki-67, EdU, or TUNEL signal in the cardiomyocytes (α-actinin positive cells). For the MI tissues, positive cells were quantified in both infarct border zone as well as remote zones.

All immunofluorescence slides were imaged at the University of Manchester Bioimaging Facility using a Zeiss AxioImager upright fluorescence microscope with EC Plan-neofluar objectives and captured using a Coolsnap HQ2 camera (Photometrics) through Micromanager software v1.4.23. Specific band pass filter sets for DAPI, FITC and Texas Red were used to prevent bleed through from one channel to the next. Images were then processed and analyzed using Fiji ImageJ ((http://imagej.net/Fiji/Downloads). The researcher was blinded during data acquisition and analysis.

Signaling pathways analysis

We generated adenoviruses carrying luciferase genes under the control of either AP1, STAT3, YAP (GAL4-TEAD), Wnt, NFκB or NFAT promoter elements. The AP1, NFκB and NFAT elements were obtained from Qiagen whereas the STAT3 element was obtained from Panomics. The luciferase reporter to monitor Hippo pathway (YAP-luciferase) was originally described in a previous publication (5). The Gal4-TEAD4 (Addgene plasmid # 24640) and the pUASluc2 (Addgene plasmid # 24343)(6) constructs were gifts from Dr Kunliang Guan and Dr Liqun Luo, respectively. The β-catenin luciferase reporter for assessing Wnt pathway was a gift from Dr Randall Moon (Addgene plasmid #12456)(7). All of the luciferase reporter constructs were cloned to adenovirus vector pAd-CMV-DEST (Invitrogen, Life Technologies) using a Gateway cloning system (Invitrogen, Life Technologies).

We used the H9c2 cardiac myoblast cell line for the screen analysis. H9c2 cells were purchased from the European Collection of Authenticated Cell Cultures (ECACC). P9-11 cells were plated in 48-well plates at a density of $2x10^5$ cells per well, along with adenovirus encoding either AP1, STAT3, YAP (GAL4-TEAD), Wnt, NFκB or NFAT luciferase

constructs. After 24 hours the media was replaced and cells were transfected with 25nM miR-411 or control using Dharmafect 1 reagent as per the manufacturer's guidelines. 48 hours post-transfection luciferase signal was detected via a Lumat LB9507 Tube Luminometer (Berthold) using a Luciferase Assay System (Promega).

Analysis of Hippo pathway regulation in cardiomyocytes

We performed sets of experiments to confirm that miR-411 also modulates Hippo/YAP pathway in primary NRCM. We used GFP-YAP construct to monitor subcellular localization of YAP. The GFP-YAP construct (pEGFP-C3-hYAP1) was a gift from Dr Marius Sudol (Addgene plasmid # 17843)(8). The GFP-YAP fragment was then cloned to an adenovirus vector using methods as described above. We then analyzed expression of YAP target genes, including Pik3cb, Birc5, Fgf2 and Tead1 in cells overexpressing miR-411 or miR control by using qRT-PCR. Finally we analyzed the expression level of proteins and phospho-proteins that are known as core members of the Hippo pathway including LATS1, phospho-LATS1, MST1, MST2, MOB1, YAP and phospho-YAP.

qPCR analysis for YAP target genes

For qPCR detecting YAP target gene expression, total RNA was isolated using Trizol reagent (Invitrogen) followed by binding, washing, and elution steps with the PureLink RNA Mini Kit (Life Technologies). 1 µg RNA was reverse-transcribed using random primers and MultiScribe Reverse transcriptase included in the High Capacity cDNA Reverse Transcription Kit (Applied Biosystems). 1 µl diluted cDNA (1:5) mixed with Brilliant III-Ultra-Fast SYBR Green qPCR Master Mix (Agilent Technologies) and primers (final concentration 1 µM forward and reverse) was used for the qPCR reaction. The cDNA reaction was run on an MJ Research PTC-200 thermal cycler (Bio-Rad), whilst qPCR reactions were loaded in triplicate in the Applied Biosystems 7500 Fast real-time PCR

system. Gene expression was determined using $2^{-\Delta\Delta Ct}$ approach(9) relative to endogenous controls (GAPDH). Primer sequences are in the tables below:

Supplemental Table 1. Primers for detecting YAP target genes in adult mouse hearts

Primer	Sequence (5' -> 3')
mCtgf-Fwd	GGGCCTCTTCTGCGATTTC
mCtgf-Rev	ATCCAGGCAAGTGCATTGGTA
mCyr61-Fwd	CTGCGCTAAACAACTCAACGA
mCyr61-Rev	GCAGATCCCTTTCAGAGCGG
mFgf2-Fwd	GCGACCCACACGTCAAACTA
mFgf2-Rev	TCCCTTGATAGACACAACTCCTC
mAnkrd1-Fwd	GCTGGTAACAGGCAAAAAGAAC
mAnkrd1-Rev	CCTCTCGCAGTTTCTCGCT
mBirc5-Fwd	GAGGCTGGCTTCATCCACTG
mBirc5-Rev	CTTTTTGCTTGTTGTTGGTCTCC
mTead1-Fwd	AAGCTGAAGGTAACAAGCATGG
mTead1-Rev	GCTGACGTAGGCTCAAACCC
mPik3cb-Fwd	CTATGGCAGACAACCTTGACAT
mPik3cb-Rev	CTTCCCGAGGTACTTCCAACT

Supplemental Table 2 Primers for detecting YAP target genes in neonatal rat cardiomyocytes:

Primer	Sequence (5' -> 3')
rCtgf-Fwd	GGGCCTCTTCTGCGATTTC

rCtgf-Rev	ATCCAGGCAAGTGCATTGGTA
rCyr61-Fwd	CTGCGCGAAGCAACTCAACGA
rCyr61-Rev	GCAGATCCCTTTCAGAGCGG
rFgf2-Fwd	GCGACCCACACGTCAAACTA
rFgf2-Rev	TCCCTTGATGGACACAACTCCTC
rAnkrd1-Fwd	GCTGGTAACGGGCAAAAAGAAC
rAnkrd1-Rev	CCTCTCGAACTTTCTCACT
rBirc5-Fwd	GAGGCTGGCTTCATCCACTG
rBirc5-Rev	CTTTCTGTTTGTTGTTGGTCTCC
rTead1-Fwd	AAGCTAAAGGTAACAAGCATGG
rTead1-Rev	GCTGATGCAGGCTCAAACCC
rPik3cb-Fwd	CTATGGCAGACACCCTTGACAT
rPik3cb-Rev	CTTCCCGGGGTACTTCCAACT

The GAPDH primers were obtained from Qiagen (Quantitect primers, Rn_Gapd_1_SG and Mm_Gapdh_3_SG).

REFERENCES

1. Mohamed TM, Abou-Leisa R, Stafford N et al. The plasma membrane calcium ATPase 4 signaling in cardiac fibroblasts mediates cardiomyocyte hypertrophy. Nat Commun 2016;7:11074.

2. Triastuti E, Nugroho AB, Zi M et al. Pharmacological Inhibition of Hippo Pathway using XMU-MP-1 Protects The Heart Against Adverse Effects during Pressure Overload. Br J Pharmacol 2019;176:3956-3971.

3. Stafford N, Zi M, Baudoin F et al. PMCA4 inhibition does not affect cardiac remodeling following myocardial infarction, but may reduce susceptibility to arrhythmia. Sci Rep 2021;11:1518.

4. Takagawa J, Zhang Y, Wong ML et al. Myocardial infarct size measurement in the mouse chronic infarction model: comparison of area- and length-based approaches. Journal of Applied Physiology 2007;102:2104-2111.

5. Tian W, Yu J, Tomchick DR, Pan D, Luo X. Structural and functional analysis of the YAP-binding domain of human TEAD2. Proc Natl Acad Sci U S A 2010;107:7293-8.

6. Potter CJ, Tasic B, Russler EV, Liang L, Luo L. The Q system: a repressible binary system for transgene expression, lineage tracing, and mosaic analysis. Cell 2010;141:536-48.

7. Veeman MT, Slusarski DC, Kaykas A, Louie SH, Moon RT. Zebrafish prickle, a modulator of noncanonical Wnt/Fz signaling, regulates gastrulation movements. Curr Biol 2003;13:680-5.

8. Basu S, Totty NF, Irwin MS, Sudol M, Downward J. Akt phosphorylates the Yes-associated protein, YAP, to induce interaction with 14-3-3 and attenuation of p73-mediated apoptosis. Mol Cell 2003;11:11-23.

9. Livak KJ, Schmittgen TD. Analysis of relative gene expression data using real-time quantitative PCR and the 2(-Delta C(T)) Method. Methods 2001;25:402-8.
